# What are the clinical signs of thiamine deficiency in elderly patients?

**DOI:** 10.1002/jgf2.479

**Published:** 2021-07-09

**Authors:** Hideki Onishi, Mayumi Ishida

**Affiliations:** ^1^ Department of Psycho‐oncology Saitama Medical University International Medical Center Hidaka City Japan

## CONFLICT OF INTEREST

The authors have stated explicitly that there are no conflicts of interest in connection with this article.

To the Editor,

We read the paper by Ohta et al. with great interest.^1^ This paper appears to be significant in that it examines the frequency of and factors related to vitamin B1 deficiency in general hospitals though a detailed study of the clinical practice undertaken by physicians, thereby clarifying the relationship with the loss of appetite.

However, in this paper, the patient selection criteria are very vague: “the presence of initial findings suggestive of vitamin B1 deficiency, assessed by general or internal medicine physicians on the day of admission based on physicians’ charts.” As no selection criteria are provided, the symptoms upon which the suspicion of thiamine deficiency is based are unclear.

We have experienced a number of cases of thiamine deficiency in cancer hospitals.^2^, ^3^, ^4^ Many patients with Wernicke's encephalopathy have also been examined, but none have demonstrated classic triad symptoms such as impaired consciousness, nystagmus, and cerebellar ataxia.^4^ We have also experienced patients who do not have any of the triad of symptoms and those who do not have a loss of appetite.^2^, ^3^ Given that previous study^5^ has shown that the symptoms of thiamine deficiency are nonspecific, it would be more useful for clinicians to provide details of the symptoms that led them to an examination of thiamine deficiency in this study.
